# Study on the Interaction between Soil and the Five-Claw Combination of a Mole Using the Discrete Element Method

**DOI:** 10.1155/2018/7854052

**Published:** 2018-08-06

**Authors:** Yuwan Yang, Mo Li, Jin Tong, Yunhai Ma

**Affiliations:** ^1^The Key Laboratory of Bionic Engineering, Jilin University, 5988 Renmin Street, Changchun 130022, China; ^2^The College of Biological and Agricultural Engineering, Jilin University, 5988 Renmin Street, Changchun 130022, China

## Abstract

A mole is a born digger spending its entire existence digging tunnels. The five claws of a mole's hand are combinative to cut soil powerfully and efficiently. However, little was known in detail about the interaction between the soil and the five-claw combination. In this study, we simulated the soil cutting process of the five-claw combination using the discrete element method (DEM) as an attempt for the potential design of soil-engaging tools to reduce soil resistance. The five-claw combination moved horizontally in the soil bin. Soil forces (draught and vertical forces) and soil failure (soil rupture distance ratio) were measured at different rake angles and speeds. Results showed that the draught and vertical forces varied nonlinearly as the rake angle increased from 10 to 90°, and both changed linearly with the speed increasing from 1 to 5 m/s. The curve of the soil rupture distance ratio with rake angles could be better described using a quadric function, but the speed had little effect on the soil rupture distance ratio. Notably, the soil rupture distance ratio of the five-claw combination in simulation was on average 19.6% lower than the predicted ratio of simple blades at different rake angles indicating that the five-claw combination could make less soil failure and thereby produce lower soil resistance. Given the draught and vertical forces, the performance of the five-claw combination was optimized at the rake angle of 30°.

## 1. Introduction

Animals have many complex and clever geometric structures that help them adapt well to diverse circumstances in nature. For example, the embossed textured surfaces on the pronotum, clypeus, and elytra of dung beetles reduce cohesion between the body and bung; the riblet structure on the skin of sharks reduces skin friction drag and makes the movement more efficient and fast. Based on bionic methods, the unique structures are utilized to design agricultural soil-engaging tools to reduce soil resistance [1, 2]. Mole rats are a completely subterranean fossorial animal and a born digger spending the entire existence in burrowing only to construct their own life systems. Their strong and powerful claws cut soil efficiently [3]. In previous articles, the geometrical characteristics of claws were studied for optimizing agricultural tillage tools and improving working efficiency. For example, Ji et al. [4] studied that the geometrical characteristics of the second claw had a significant soil-cutting performance. Li et al. [5] showed that a biomimetic disc designed based on the profile curves of the second claw performed better in structural strength and cutting efficiency using finite element analysis. Tong et al. [6] found out that the torque of the optimal biomimetic blades designed based on the geometries of the tips of claws was lower during soil-rototilling and stubble-breaking operations compared with the conventional universal blade. As is well known, the mole has evolved into the structure of five-claw combination to self-adapt to its living circumstances. The five claws work together to complete the digging task. In this study, the interaction between soil and five-claw combination was investigated with the aim of further optimizing soil-engaging tools for minimizing energy consumption.

**Figure 1:**
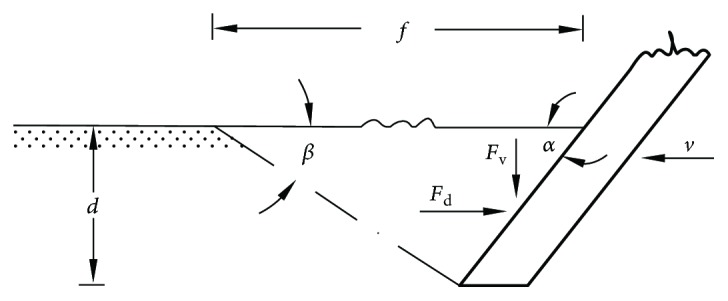
Soil failure and soil forces according to Wheeler and Godwin [30].

During the interaction between soil and tools, soil failure is intentionally caused by the applied force of tools [7]. The soil rupture distance ratio (*m*) is an important parameter to characterize the soil failure. It was defined as the ratio of the rupture distance of blade (*f*) on the soil surface to the working depth (*d*) (see Figure 1) according to Godwin and Spoor [8], and it was presented as

(1) $$\begin{array}{c} m=\frac{f}{d}. \end{array}$$

The soil rupture distance ratio of blades and tines had been studied as influenced by different rake angles, respectively [8, 9]. Soil forces of blades and tines including draught (*F*_d_) and vertical (*F*_v_) forces (see Figure 1) have been predicted using the passive earth pressure theory [8, 10–12]. However, the analytical models are not suitable to complicated models. Also, the empirical method is costly and time-consuming. As a consequence, numerical methods such as finite element method (FEM) and discrete element method (DEM), which save the experiment time and cost and also can solve the complex situations, have been successfully applied to the analysis of the soil-tool interactions [13, 14]. FEM is excellent for continuum analysis; while soil deformation involves the formation of cracks, the movement of soil particles and separation and mixture of soil layers are difficult to simulate [15]. Conversely, DEM is particularly suitable to model the soil deformation and forces of soil-tool interactions, and it may serve as a predictive simulation tool in the process of designing the tillage tool's shape [16].

Accurate simulation of soil failure depends on defining and calibrating the soil particle model. Ucgul et al. [17] used a hysteretic spring (plastic) contact model (HSCM) for a sweep tool operated in a cohesionless soil with a good correlation between the predicted and measured tillage forces for both draught and vertical forces (*R* ^2^ = 0.95–0.99). The model parameters were calibrated by the angle of repose test and penetration test. Then a linear cohesion model was integrated with the HSCM to model the plastic and cohesive behaviors of soil and its interaction with a tillage tool by Ucgul et al. [18–20]. Direct shear tests were used to calibrate the model parameters [19]. The good correlations give confidence to recommend further investigation of the use of the hysteretic spring contact model for a wider range of soil conditions and types of tillage tools [18]. In this study, the DEM simulation model studied by Ucgul et al. would be considered to simulate the soil failure and predict the soil force of the structure of the multiclaw combination of a mole.

Here, the soil forces and soil failure of the five-claw combination of a mole were studied during the interaction with soil using the discrete element method. The aim was to investigate the possibility of applying the structure of a five-claw combination to agricultural soil-engaging tools to minimize energy consumption.

## 2. Materials and Methods

### 2.1. Model Five-Claw Combination

#### 2.1.1. Description of Five-Claw Combination of a Mole

**Figure 2:**
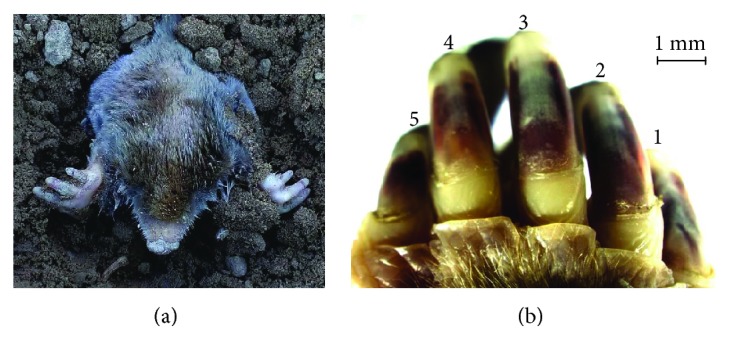
(a) A mole and (b) the five-claw combination.

**Figure 3:**
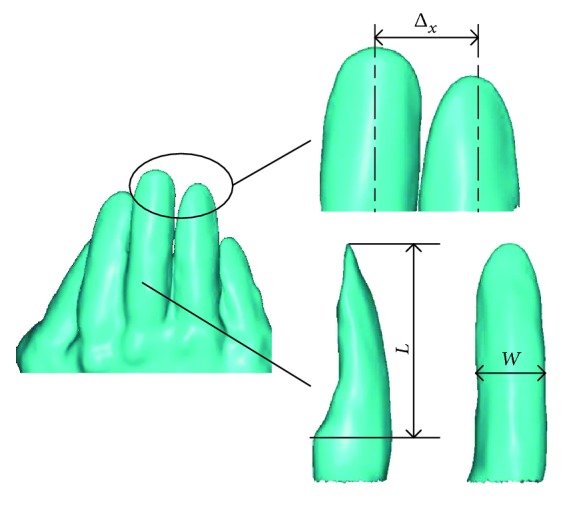
The point cloud of the five-claw combination.

The mole rats (*Scaptochirus,* Talpidae) were obtained in the northeast of China where they are most common and inhabit mostly underground. Their broad and strong hands which consist of five different claws (see Figure 2) were scanned by a three-dimensional laser scanner (Handyscan700, Creaform, Canada), and the point cloud (see Figure 3) was created with the reverse engineering software program of ImageWare (version 13, Siemens PLM software, Germany). After a series of procedures, such as smoothing, reducing, and simplification, the five-claw combination was reconstructed into a surface, and then it was generated as an entity from a surface on SolidWorks software. It was considered as 5 times the size of the prototype which was too small to model. The parasolid text format was saved and imported to the EDEM™ software for simulation. The horizontal movement of the five-claw combination in soil was evaluated as this movement is common in soil-engaging tools.

#### 2.1.2. Characteristics of the Five-Claw Combination

Every hand of a mole has five different claws. From Figures 2(b) and 3(a), the 3rd claw was considerably longer than the 1st, 2nd, 4th, and 5th claws with the 5th claw being very small. The length (*L*) and width (*W*) of each claw were measured and displayed in Table 1. The ratio of width to length of each claw was calculated. It was found out that all the ratios of the main three claws (i.e., the 2nd, 3rd, and 4th claws) were near 0.3, while the other two claws (i.e., 1st and 5th claws) with larger ratios indicated their thinness and weakness, which proved again that the middle three claws played the main roles in digging. Moreover, the five-claw combination always engages soils with a spaced arrangement. Thereby, the interval (Δ) between two adjacent claws (Figure 3) was also an important parameter to determine the structure of the five-claw combination. They were measured for characterizing the structure of five-claw combination (Table 1). It was observed that the middle three claws were arranged more closely than the other two claws of 1st and 5th claws. In our study, the model of five-claw combination would present these characteristics.

### 2.2. EDEM Simulations

The DEM was undertaken using EDEM 2.7 software. Soil particles were represented by spherical particles with a 4 mm radius particle size which was selected to reduce the computation time. The particle size was randomly generated in the range of 0.95–1.05 times the 4 mm size. Particles were confined in a soil bin constructed by five EDEM walls. The dimensions of the soil bin were 400 mm long × 200 mm wide × 150 mm deep, which allowed the five-claw combination to have enough distances to avoid any edge effects from the soil bin walls. The total number of soil particles produced was 10,000. The final bulk density of the particles was 1283 kg/m^3^. A linear cohesion integrated hysteretic spring contact model suggested by Ucgul et al. [18–20] was used to model the cohesive behavior of soil. The material of the claws was considered as steel-like tillage tools. A Hertz-Mindlin contact model (HMCM) was used to model the interaction between soil and claws. The model assumed a nonlinear elastic manner to predict the behavior between soil and five-claw combination. All related parameters are shown in Table 2.

**Figure 4:**
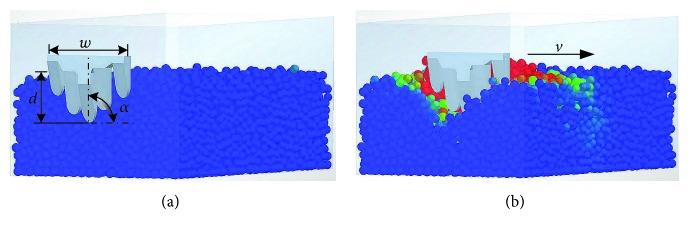
Interaction of soil and the five-claw combination: (a) at the initial state; (b) travelling in the soil bin. *w* = blade width; *d* = working depth; *α* = rake angle; *v* = working speed.

The five-claw combination was positioned at the end of the soil bin at a specified rake angle and working speed before the model travelling (see Figure 4(a)). The working depth was fixed at 45 mm in order to assure claws interact with the soil particles as fully as possible. To investigate the effects of the rake angle (*α*) and working speed (*v*) on the soil forces and soil failure, the simulations were run for rake angles from 10 to 90° at 20° intervals and working speeds from 1 to 5 m/s at an interval of 1 m/s. When the working speed was varied, the rake angle was kept constant (*α* = 90°). When the rake angle was varied, the working speed was kept constant (*v* = 3 m/s). Each simulation was repeated three times as there was always a variation in results, and the average value of the simulation results was taken as the final result. The five-claw combination interacted with soil particles as it traveled (see Figure 4(b)), and the resultant soil forces and soil failure were conducted as described in the following section.

### 2.3. Calibration of the Model Soil Particles

Calibration was done through the simulation and matching of the soil rupture distance ratio (*m*) with the values predicted by an analytical method. This similar approach has been used by Mak et al. [21] and Li et al. [22] for calibrating a PFC model. The blade was a wall with a width of 75 mm and traveled at a speed of 3 m/s with a working depth of 45 mm through the soil particles model. The rake angles of 30°, 50°, 70°, and 90° were used in the calibration. The soil rupture distance ratio (*m*) was calculated according to (1). The soil rupture distance of soil failure (*f*) made by the blade travelling was measured on the soil surface. The soil rupture distance ratio was compared with the prediction of the values by Hettiaratchi et al. [9]. The soil particles model parameters were confirmed when these simulation results matched with the prediction by Hettiaratchi et al. [9].

### 2.4. Data Collection and Processing

**Figure 5:**
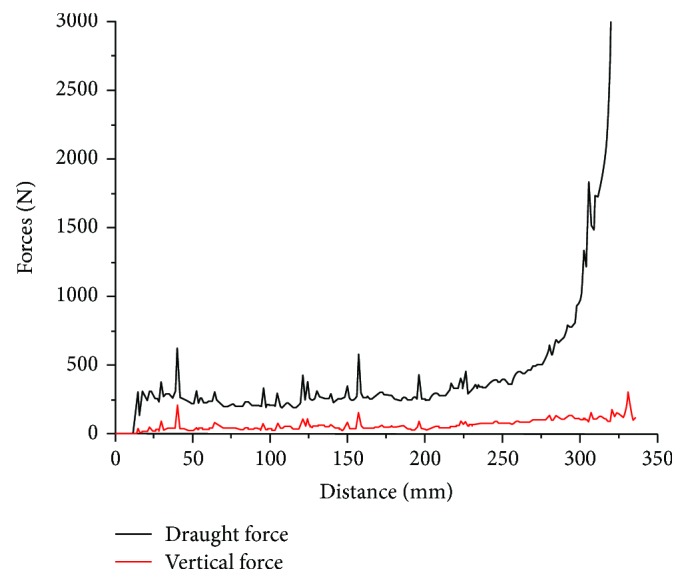
An example of force curves from the simulation. Working speed = 3 m/s; rake angle = 90°.

During the travelling of five-claw combination in the soil bin, the draught and vertical forces were monitored over the length of the soil bin. The forces fluctuated due to the random nature of soil particle disturbance (see Figure 5). The draught and vertical forces increased when the five-claw combination began to contact with the soil particles and then fluctuated around a constant value when the five-claw combination advanced through the soil particles. The average values of draught and vertical forces were taken over the constant section of the force curve (corresponding to the midsection of the soil bin between 50 and 250 mm).

**Figure 6:**
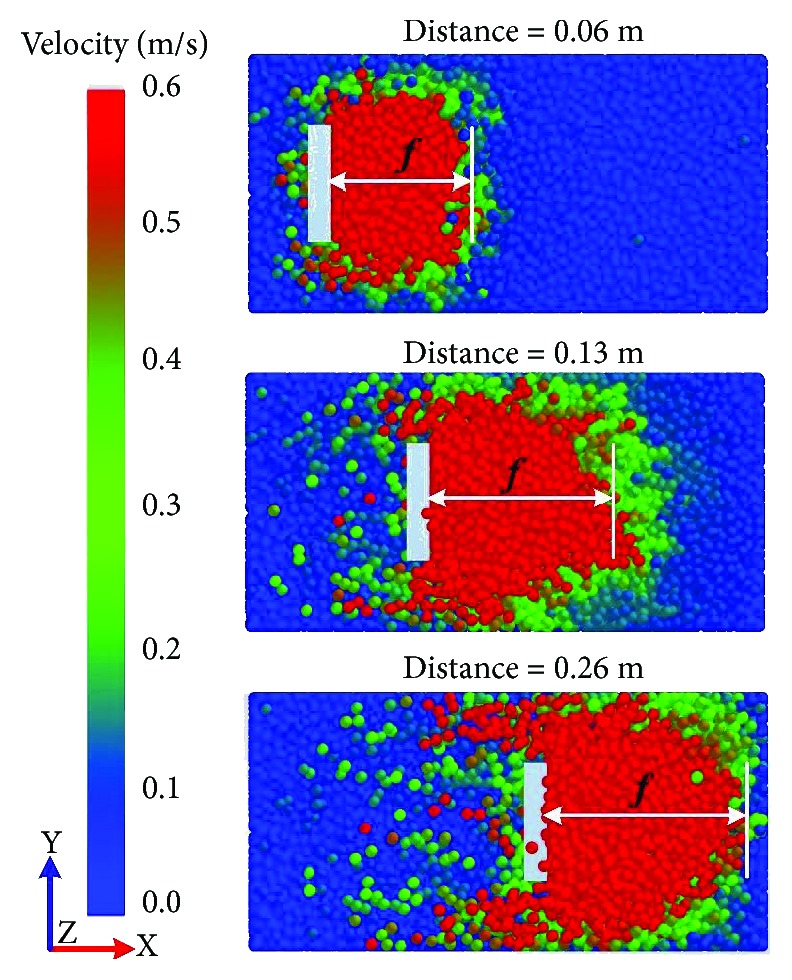
Snapshots of the top view of soil failure during the simulations of the velocity field. *f* = soil rupture distance; red zone = soil failure region.

Snapshots of the top view taken during the simulations of the velocity field are presented in Figure 6. The different color levels presented the different velocities of soil particles. The red color meant the larger velocity of soil particles, the green color meant very small velocity of soil particles, and the blue color meant that the state of soil particles was static. The soil failure boundary was mainly in the section of the red zone. The soil rupture distance (*f*) was the maximum longitudinal distance from the model surface to the front of soil failure boundary (see Figure 1). Three stages (i.e., original stage, middle stage, and end stage) during the travelling of five-claw combination were displayed in Figure 6. It was suggested that the soil rupture distance at the original stage of the simulation was obtained to be the final result which was consistent with the study by Shmulevich et al. [23]. Then the soil rupture distance ratio (*m*) was calculated according to (1).

## 3. Results and Discussion

### 3.1. Soil Particles Model Calibration Results

**Figure 7:**
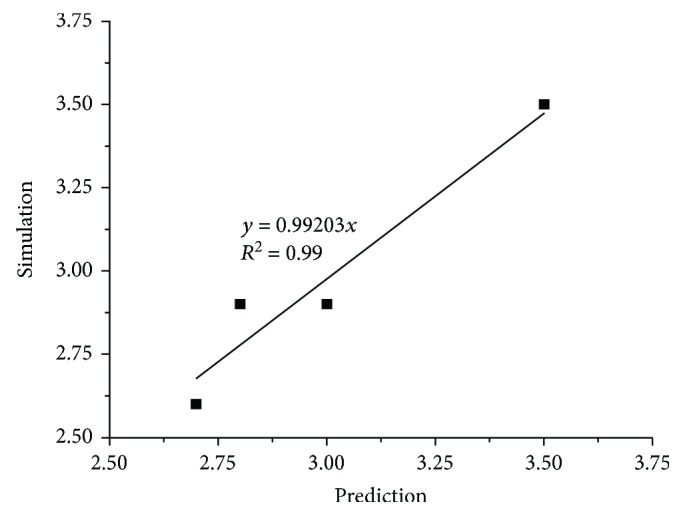
The correlation of the soil rupture distance ratio (*m*) between the simulation and prediction.

The soil rupture distance ratio (*m*) of simulations was matched with the corresponding values predicted by Hettiaratchi et al. [9] at different rake angles in Figure 7. The simulated results slightly underestimated the soil rupture distance ratio of prediction (*y* = 0.99203*x*). However, the value of the coefficient of determination (*R* ^2^ = 0.99) showed that the examined data pairs were close to the correlated line. Furthermore, the average error of the soil rupture distance ratio in simulation compared with the corresponding values of prediction at different rake angles was −3%; therefore, the simulation model of soil particles behaved fairly well regarding the estimation of the soil rupture distance ratio at different rake angles. Ucgul et al. [18–20] also recommended that the DEM simulation parameters had good potential to model tillage forces in a range of soil and operating conditions.

### 3.2. Soil Forces Affected by Rake Angles

**Figure 8:**
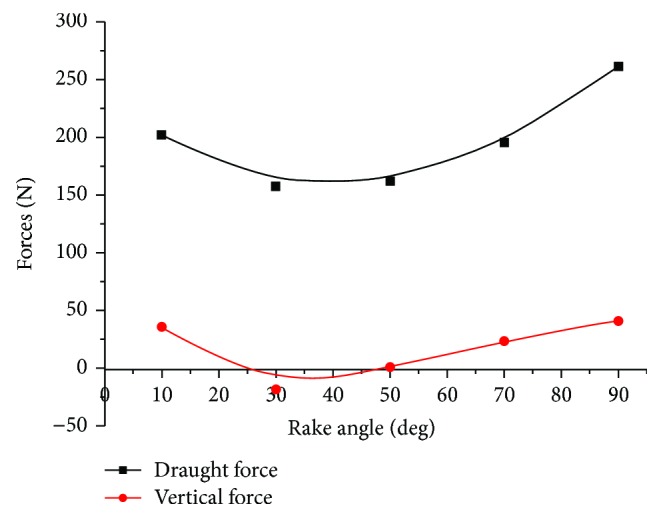
The draught and vertical forces of the five-claw combination affected by the rake angle.

The draught and vertical forces of five-claw combination affected by rake angles are shown in Figure 8. On average, the draught forces were 12 times the magnitude of vertical forces. All the draught and vertical forces varied nonlinearly with the rake angle in the range of 10 to 90°. All forces first decreased with the rake angle increasing from 10 to 30°, and then increased from 30 to 90°. Thereby, the draught forces and vertical forces all minimized at the rake angle of 30°. Interestingly, the vertical forces were negative at the rake angle of 30°. Godwin [24] summarized that the draught and vertical forces of the tine were affected by rake angles of 22.5 to 112.5° and found out that the draught and vertical forces increased with rake angle. He stated that there was a crossover value for the vertical forces upward to downward force at the critical rake angle of 67.5°. The negative vertical forces meant that the tools had a noticeable behavior of penetrating into the soil. Therefore, the five-claw combination had a better penetration performance at the rake angle of 30°. Overall, the rake angle of 30° was the optimal operating condition for the five-claw combination to produce lower draught forces and better soil penetration performance. This rake angle was also recommended by Li et al. [22] who studied the effects of rake angles of a bear claw on the soil cutting forces.

**Figure 9:**
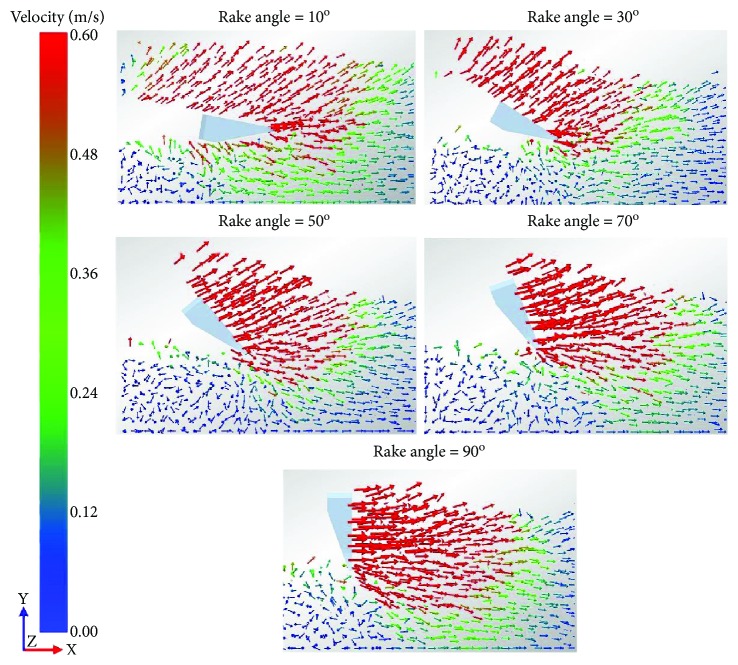
Velocity field in the vicinity of the five-claw combination at different rake angles.

Soil flow in the vicinity of the five-claw combination at different rake angles was observed in the simulation results, as presented in Figure 9. The simulation results of the velocity field of soil particles were schematically described. The velocity of each particle was marked by an arrow, of which, the length and direction indicate the magnitude and direction of the velocity, respectively. At the rake angle of 10°, many soil particles that moved forward and upward existed above the five-claw combination. The largest velocity which was corresponding to the longest arrow appeared in front of the five-claw combination. Also, many soil particles moving forward and downward appeared under the five-claw combination. As a result, the five-claw combination would suffer larger crowding and soil gravity. Therefore, the draught and vertical forces of the five-claw combination were very large as described in Figure 8 at the rake angle of 10°. At the rake angle of 30°, the number of soil particles moving forward and upward above the five-claw combination declined. Also, a smaller number of soil particles under the five-claw combination moved forward and downward, which meant the five-claw combination would not compact the tillage pan and could better penetrate into soils. Thus, the vertical forces of the five-claw combination were negative and the draught forces were weakened. Then, with the increasing rake angle, the number of disturbed soil particles increased and the velocity gradually increased. Evidently, at the rake angle of 90°, the number of soil particles at larger velocity maximized and the region of evenly-disturbed soil particles expanded to the bottom of the five-claw combination. Overall, the variation trend of draught and vertical forces observed in Figure 8 was in parallel with the situation of soil flow in the vicinity of the five-claw combination.

### 3.3. Soil Forces Affected by Speeds

**Figure 10:**
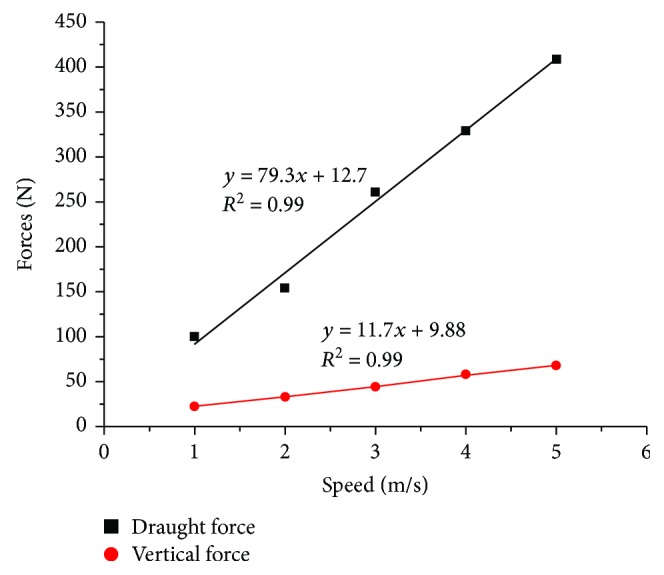
The draught and vertical forces of the five-claw combination affected by speed.

The draught and vertical forces of the five-claw combination affected by speeds are displayed in Figure 10. On average, the draught forces always surpassed the vertical forces. The fitted curves indicated the draught and vertical forces were enlarged linearly when the speed increased from 1 to 5 m/s. But the variation rate of draught forces against speeds was larger than that of vertical forces. The two coefficients of determination (*R* ^2^) of the fitted curves both reached 0.99, which meant the data were close to the correlated line. This variation trend accorded with the relationship of draught and vertical forces of tine and the speed in the range of 0 to 12 km/h which was summarized by Godwin [24].

**Figure 11:**
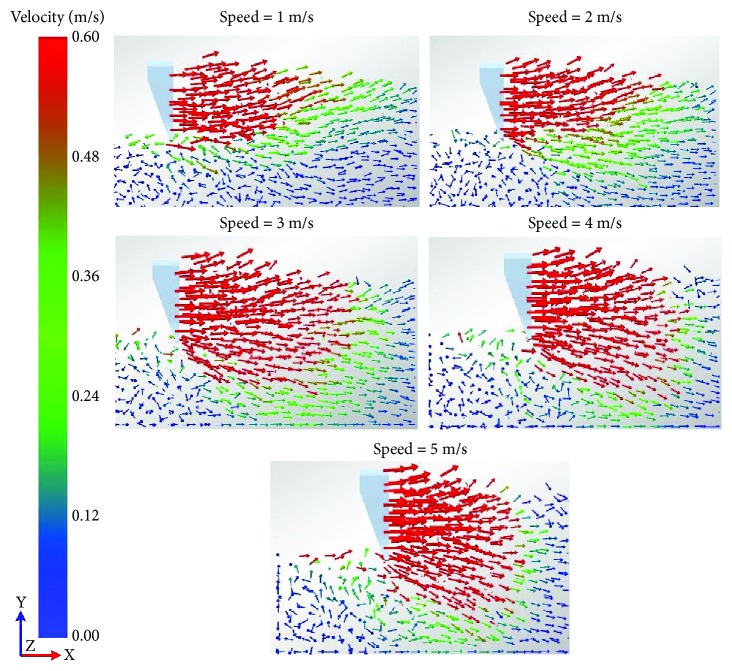
Velocity field in the vicinity of the five-claw combination at different speeds.

The soil flows in the vicinity of the five-claw combination at different speeds were observed in the simulation results as shown in Figure 11. At the speed of 1 m/s, a smaller number of soil particles moved forward and upward in front of the five-claw combination. The largest velocity mainly appeared on the soil surface. Fewer soil particles moved forward and downward under the five-claw combination. Thus, the draught and vertical forces of the five-claw combination were weakened at the speed of 1 m/s as described in Figure 10. Then with the increasing speed, the number of disturbed soil particles increased and the velocity was gradually accelerated. At the speed of 5 m/s, the number of soil particles at larger velocity maximized and mainly assembled in front of the five-claw combination. Even the region of disturbed soil particles expanded to the bottom of the five-claw combination. Therefore, the draught and vertical forces were enhanced rapidly as shown in Figure 10.

### 3.4. Soil Rupture Distance Ratio Affected by Rake Angle

**Figure 12:**
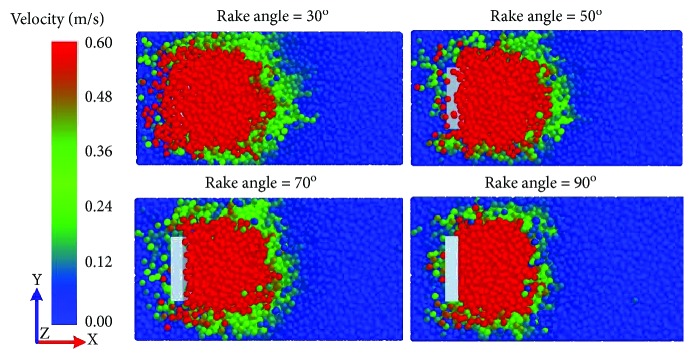
Soil failure of the five-claw combination in the top view of the velocity field at different rake angles.

**Figure 13:**
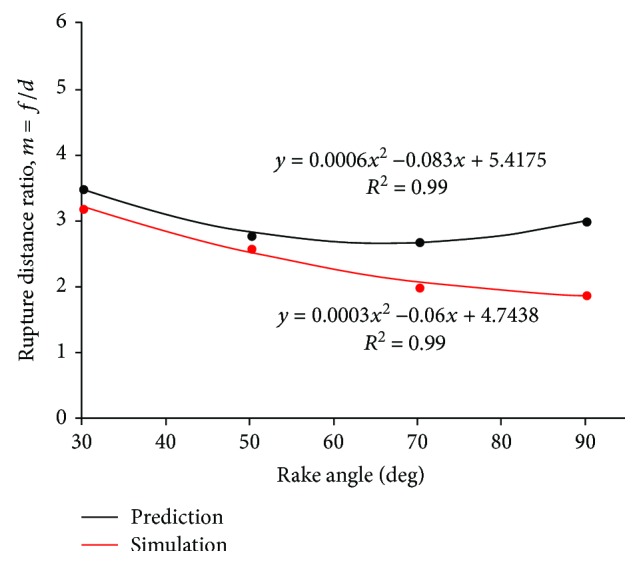
The soil rupture distance ratio of the five-claw combination affected by rake angle.

Figure 12 shows the variation of soil rupture distance with rake angle. The red zone of the velocity field was narrowed down in the front of the five-claw combination. Since the soil rupture distance ratio (*m*) was proportional to the soil rupture distance (*f*) according to (1), the variation trend of soil rupture distance with rake angles meant the soil rupture distance ratio would diminish with rake angles. It was calculated and shown in Figure 13. The soil rupture distance ratio was significantly affected by the rake angle and described nonlinearly as the rake angle rose from 30 to 90°. This nonlinear trend was well described by a quadratic function with a coefficient of determination (*R* ^2^) of 0.99.

**Figure 14:**
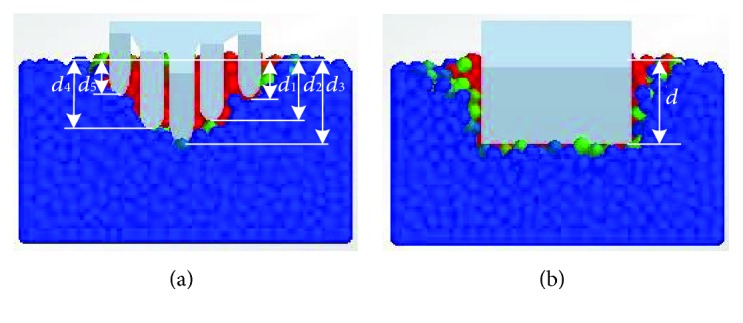
The comparison of the working depth between the five-claw combination and the blade in the soil-cutting process: (a) the five-claw combination working at varying depths (*d*_1_, *d*_2_, *d*_3_, *d*_4_, and *d*_5_ are the working depths of the corresponding claw, resp.); (b) the blade working at a fixed depth (*d* is the working depth of the blade).

This variation trend underlying the soil rupture distance ratio of the five-claw combination affected by rake angles was similar to the study of simple blades by Hettiaratchi et al. [9]. The corresponding predictions of blades were also displayed in Figure 13. Generally, the soil rupture distance ratio of the bionic model was about 19.6% lower than the predicted values of simple blades (see Table 3). When the rake angle was below 50°, the error of the soil rupture distance ratio of the five-claw combination was smaller than the corresponding predicted values of simple blades, but the error was gradually enlarged when the rake angle increased from 50 to 90°. It was implied the soil failure was affected significantly by the structure of five-claw combination. The five-claw combination would create less soil failure due to the five claws working at varying depth in the soil-cutting process (see Figure 14) and thereby got lower soil forces. The force reducing behavior by the structure of five-claw combination was prominent at large rake angles, which should be further studied though. Overall, the structure of five-claw combination is potentially applicable to agricultural tillage implements, aiming to minimize energy consumption by changing soil failure.

### 3.5. Soil Rupture Distance Ratio Affected by Speed

**Figure 15:**
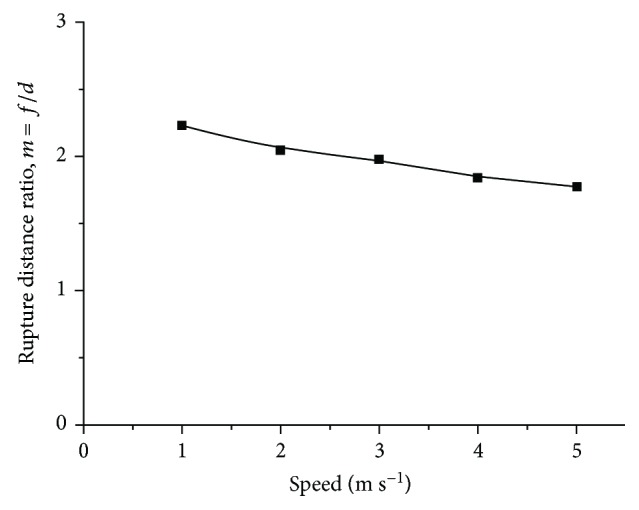
The soil rupture distance ratio of the five-claw combination affected by speed.

**Figure 16:**
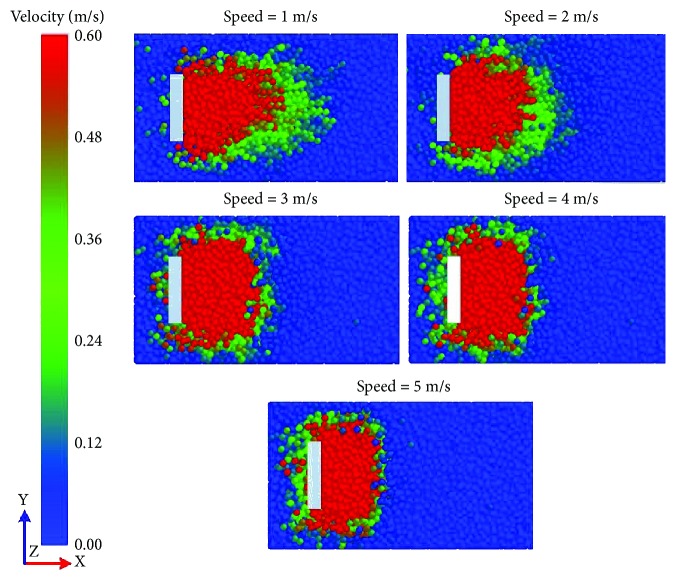
Soil failure of the five-claw combination in the top view of the velocity field at different speeds.

Figure 15 shows that the soil rupture distance ratio of the five-claw combination was affected by speed. It decreased from 2.23 to 1.78 as the speed increased from 1 to 5 m/s, which could be found from the soil rupture distance with speeds in Figure 16. The red zone of the velocity field was narrowed down but concentrated in front of the five-claw combination. Stafford [25] studied the effect of speed on soil shear strength and found out that it is difficult to investigate the variations of soil cohesion and internal friction angle with speeds. Thus, soil properties changed in a complicated way as influenced by speeds, which led to the variation of the soil rupture distance ratio.

### 3.6. Possible Application of the Structure of the Five-Claw Combination

In tillage operations, the big problems to be solved urgently are the larger soil resistance and higher energy consumption. The structure of the five-claw combination of a mole could diminish the soil rupture distance ratio and thereby help to reduce soil forces, while it is different with other methods by varying the working operations (e.g., vibrating tillage tools [26] and reverse-rotary tiller [27]) or by minimizing penetration resistance (optimizing the cutting edges of tillage tools [28, 29]). Actually, the deeper working central tine and shallower working wings are already used commercially and this work validates their use. Therefore, the structure of the five-claw combination is potentially applicable to soil-engaging tools, such as a plough, subsoiler, and rotary tiller blade.

## 4. Conclusions

The interaction between soil and the five-claw combination was simulated using the discrete element method for studying the soil forces and soil failure of the five-claw combination of a mole. Simulation showed the following:

1. The draught and vertical forces of the five-claw combination were nonlinearly affected by rake angles. Particularly, the draught forces were reduced and the soil penetration performance was improved at the rake angle of 30°. And the draught and vertical forces both increased linearly as the speed rose from 1 to 5 m/s.
2. The soil rupture distance ratio changed with the rake angle in a nonlinear trend, which was well fitted by a power function with a coefficient of determination of 0.99. On average, the soil rupture distance ratio of the five-claw combination was 19.6% lower than the corresponding values of simple blades by Godwin and Spoor [8] and was decreased from 2.23 to 1.78 as the speed rose from 1 to 5 m/s.

Overall, the structure of the five-claw combination with varying depth of operation plays an important role in reducing soil resistance through decreasing the soil rupture distance ratio. This study provides a novel geometry for designing soil-engaging tools with less energy consumption. Of course, the effect of the soil type and condition on the interaction between soil and the five-claw combination needs further study.

## Figures and Tables

**Table 1:** Characteristics of the five-claw combination.

Claw	*L* (mm)	*W* (mm)	*W*/*L*	Interval between two adjacent claws	*Δ* (mm)
1st	6.47	2.34	0.362	1st and 2nd	3.86
2nd	7.83	2.42	0.309	2nd and 3rd	2.79
3rd	8.82	2.586	0.293	3rd and 4th	3.04
4th	7.99	2.46	0.308	4th and 5th	3.12
5th	5.43	2.06	0.380		

**Table 2:** DEM parameters used in the simulations.

Property	Value
Density of soil particles (kg/m^3^)	2600 [31]
Density of steel (kg/m^3^)	7820 [32]
Shear modulus of soil (Pa)	5 × 10^7^ [33]
Shear modulus of steel (Pa)	7.9 × 10^10^ [32]
Yield strength of the soil (Pa)	5.88 × 10^5^ [17]
Poisson's ratio of soil	0.3 [33]
Poisson's ratio of steel	0.3 [32]
Coefficient of restitution of soil-soil	0.6 [31]
Coefficient of restitution of soil-steel	0.6 [31]
Coefficient of friction of soil-soil	0.57 [17]
Coefficient of friction of soil-steel	0.5 [17]
Coefficient of rolling friction of soil-soil	0.16 [17]
Coefficient of rolling friction of soil-steel	0.05 [17]
Cohesive energy density between soil-soil (J/m^3^)	5000 [19]
*n*_*k*_—the stiffness factor	0.95 [17]
*n*_*c*_—the damping factor	0.05 [17]

**Table 3:** Comparison of the soil rupture distance ratio by simulation of the five-claw combination and prediction of simple blades by Hettiaratchi et al. [9].

Rake angle (deg)	Soil rupture distance ratio (*m*)		Error (%)	Average error (%)
	Simulation	Prediction		
30	3.2	3.5	−8.6	−19.6
50	2.6	2.8	−7.1	
70	2	2.7	−25.9	
90	1.9	3	−36.7	

## Data Availability

The raw data used to support the findings of this study are included within a supplementary information file.

## Supplementary Materials

Supplementary 1 Table S1: an example of force curves from the simulation (working speed = 3 m/s; rake angle = 90°). The draught and vertical forces recorded along the travelling distance of the five-claw combination during the interaction with soil at the speed of 3 m/s and rake angle of 90°.

Supplementary 2 Table S2: calibration of the model soil particles. The soil rupture distance ratio (*m*) of a blade with the values predicted by the analytical method from Hettiaratchi et al. [9]. The corresponding values of the soil rupture distance ratio (*m*) obtained from the simulation.

Supplementary 3 Table S3: the draught and vertical forces affected by rake angles. The draught and vertical forces recorded along the travelling distance of the five-claw combination during the interaction with soil at the rake angles from 10° to 90° at 20° intervals and working speed of 3 m/s.

Supplementary 4 Table S4: the draught and vertical forces affected by speeds. The draught and vertical forces recorded along the travelling distance of the five-claw combination during the interaction with soil at the working speeds from 1 to 5 m/s at an interval of 1 m/s and rake angle of 90°.

Supplementary 5 Table S5: the soil rupture distance ratio affected by rake angles. The soil rupture distance ratio (*m*) obtained from the simulation at the rake angles of 30°, 50°, 70°, and 90°.

Supplementary 6 Table S6: the soil rupture distance ratio affected by speeds. The soil rupture distance ratio (*m*) obtained from the simulation at the speeds of 1, 2, 3, 4, and 5 m/s.

Supplementary 7 Figure S7: statistical analysis of the correlation of the soil rupture distance ratio between the simulation and prediction. The soil rupture distance ratio (*m*) of simulations matched with the corresponding values predicted by Hettiaratchi et al. [9] at different rake angles.

Supplementary 8 Figure S8: statistical analysis of the draught and vertical forces of the five-claw combination affected by rake angles. The draught and vertical forces varied with the rake angle in the range of 10° to 90°.

Supplementary 9 Figure S9: statistical analysis of the draught and vertical forces of the five-claw combination affected by speeds. The draught and vertical forces varied with the speed increasing from 1 to 5 m/s.

Supplementary 10 Figure S10: statistical analysis of the soil rupture distance ratio of five-claw combination affected by rake angles. The soil rupture distance ratio (*m*) of simulations and predictions varied with the rake angle in the range of 30° to 90°.

Supplementary 11 Figure S11: statistical analysis of the soil rupture distance ratio of the five-claw combination affected by speeds. The soil rupture distance ratio (*m*) of simulations and predictions varied with the speed in the range of 1 to 5 m/s.
